# Effect of reward on electrophysiological signatures of grid cell population activity in human spatial navigation

**DOI:** 10.1038/s41598-021-03124-y

**Published:** 2021-12-08

**Authors:** Wenjing Wang, Wenxu Wang

**Affiliations:** School of Systems Science, Beijing Normal University, Beijing, 100875 China

**Keywords:** Cognitive neuroscience, Learning and memory

## Abstract

The regular equilateral triangular periodic firing pattern of grid cells in the entorhinal cortex is considered a regular metric for the spatial world, and the grid-like representation correlates with hexadirectional modulation of theta (4–8 Hz) power in the entorhinal cortex relative to the moving direction. However, researchers have not clearly determined whether grid cells provide only simple spatial measures in human behavior-related navigation strategies or include other factors such as goal rewards to encode information in multiple patterns. By analysing the hexadirectional modulation of EEG signals in the theta band in the entorhinal cortex of patients with epilepsy performing spatial target navigation tasks, we found that this modulation presents a grid pattern that carries target-related reward information. This grid-like representation is influenced by explicit goals and is related to the local characteristics of the environment. This study provides evidence that human grid cell population activity is influenced by reward information at the level of neural oscillations.

## Introduction

Grid cells have a periodic equilateral triangle firing pattern, which is considered the basis for their metric measurement in spatial navigation^1^. Grid cells with different scales of firing fields can provide accurate positioning information through scale combination^2^. This internal metric helps organisms achieve path integration and vector-based navigation behaviors such as homing in the absence of external information^3^. Since it is the universal metric system for spatial navigation, the grid pattern should be rigid and immutable. However, based on the in-depth study of grid cells, the grid pattern is not strictly unchanged but can be squeezed, stretched, deformed or even discretely broken under the effects of various types of environmental spatial information^4–6^ and can also be merged globally after multiple subenvironmental spaces are connected^7^. This result is a step forward in the understanding of grid cells, which provide flexible metric systems that adapt to the spatial environment and assist organisms in completing path integration in complex spatial environments. Recently, some breakthrough studies on the information types coded by grid cells have been published. In 2016, Tim Behrens et al. found that grid cells also present a typical hexadirectional modulation mode when encoding abstract bird space with nonspatial information^8^. In the same year, Neil Burgess and his team also discovered the hexadirectional modulation of grid cells for the encoding of imaginary space^9^. Afterward, the discussion and research on nonphysical spatial information (such as conceptual space, social space, etc.) encoded by grid cells were initiated^10,11^. Thus, the grid cells in the entorhinal cortex appear to encode a broader “cognitive map” in a more general sense, with their distinctive equilateral triangular periodicity patterns^12^. Since grid cells can encode both spatial and nonspatial information, will they be affected by nonspatial information when encoding the physical environment? Boccara et al. and Butler et al. independently conducted experiments on rodents to address this question in 2019^13,14^. The results of both studies show that the grid pattern is indeed affected by nonspatial information such as goal reward, resulting in local deformation and structural adjustment. For the exploration of nonspatial information encoded by grid cells, particularly high-level decision-making information such as navigation goals, the more important research object is humans, but experimental research on humans is very scarce. Limited by experimental conditions, research on changes in the human grid pattern is often limited to noninvasive functional magnetic resonance imaging (fMRI) measurements, and the signal from the entorhinal cortex in the limbic system where grid cells are located is difficult to record. Here, we performed a VR desktop navigation experiment on patients with medically intractable epilepsy and recorded EEG data from deep electrodes in the entorhinal cortex to investigate the nonspatial effect of grid cell population activity. Based on recent research showing that theta oscillations in the human entorhinal cortex carry hexadirectional modulation information of grid cells^15,16^, we also used the same method to study the changes in grid cell population activity by measuring theta power. The hexadirectional modulation of theta power, which reflects the regular activity of grid cells, only appears in the experimental stage without an effect of a specific goal object. However, when a clear goal object is present, the hexadirectional modulation of theta power disappears, indicating that the grid pattern is disturbed by the target reward. In addition, the disturbance is also affected jointly by environmental boundaries, and in the central region lacking boundary anchor cues, it will depend more on the self-centered localization function of grid cells. Our study is the first to analyse the effect of nonspatial information on a grid pattern in human spatial navigation by recording EEG signals from intracranial electrodes. This study provides mesoscopic evidence for exploring the underlying coding patterns of the human entorhinal cortex and the coupling effect of spatial and nonspatial factors on the entorhinal cortex and advances the research on the multidimensional generalized functional framework of grid cells.

## Material and methods

### Participants in the task

Electroencephalograms were recorded from stereotactically implanted electrodes in patients with medically intractable epilepsy, and their seizure foci were located to guide their respective treatment. The mean age of the 9 patients (3 females) was 27.1 years (SD = 8.2).

### Ethics approval

The Scientific Research Ethics Committee of Beijing Normal University approved all procedures performed in this study. Written informed consent was obtained from all patients. All the experiments were performed in accordance with relevant guidelines and regulations.

### Spatial memory task

**Figure 1:**
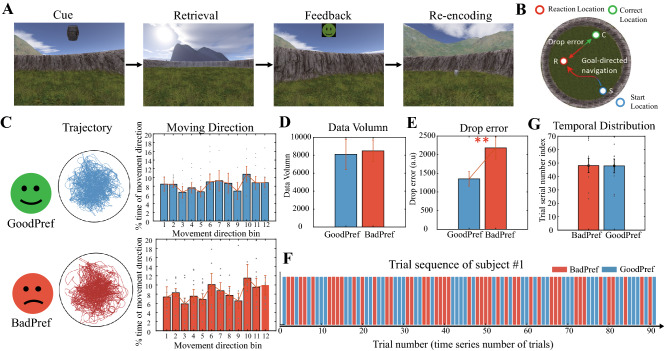
Paradigm and behavioral division with the effect of goals. (**A**) The four task stages of each trial in the memory task performed by the participants (adapted from Chen et al. (Current Biology, 2018)). (**B**) An aerial view of the circular arena. The drop error is defined as the relative distance between the subjective response location and the reallocation. (**C**) Two types of groups are defined by drop error in behavior. The top row is GoodPerf, and the bottom row is BadPerf. The middle column is the trajectory of the participants, achieving extensive coverage in both groups. The right-most column shows the movement direction of the participants, which is evenly distributed at all angles between the two groups (Rayleigh’s tests for non-uniformity, *p* > 0.05). (**D**) Comparison of experimental data between GoodPerf and BadPerf. A significant difference was not observed between the two groups (*t* test, *p* > 0.5). (**E**) Comparison of performance in the trials corresponding to GoodPerf and BadPerf goal objects. The difference in drop error between the two groups was significant (*t* test, *p* < 0.001). (**F**) An example of the time sequence of GoodPref and BadPref trials during the whole experiment period (taking subject #1 as an example), and the trial sequence number index of each type group is calculated based on the average sequence number of all trials in this category. (**G**) Temporal distribution of the two groups in the experiment. We define the trial serial number index to represent the temporal distribution, represented by the average serial number of all trials in each group in the experiment. Statistics showed that Goodpref and Badpref were evenly distributed in the experiment, without significant difference (paired *t* test, *p* < 0.05).

The participants navigated freely in a circular virtual arena adapted by Doeller et al.^17^ using a laptop to perform a task of remembering the location of goal objects that included rewards. The environment consists of a meadow plane (9500 virtual units in diameter) surrounded by a wall with a circular boundary. The navigation task paradigm was written using UnrealEngine 2 (Epic Games). During the initial learning phase at the start of the experiment, patients were asked to remember the locations of eight different everyday objects. For about 10 min, participants will traverse the real locations of the eight objects they will see in the experiment one by one, picking them up according to the objects presented in the scene. Patients then completed different numbers of trials. Each trial consisted of cue, retrieval, feedback, and recoding phases (Fig. 1A). During the cue phase, the participant viewed one of the objects (for 2 s). During the retrieval phase, they used the arrow keys (left, right, and forward) on the laptop keyboard to navigate to the location of the relevant object. The duration of this phase is a self-determined step. When the participants reached what they thought was the correct position, they pressed the space bar to locate the object (Fig. 1B). Depending on the accuracy of the response, the patient received feedback from one of five possible cartoon faces (lasting 1.5 s). The more red and sad the cartoon face was, the greater the error in behavior. The actual reward received by the participants after completing the experiment will be given according to the behavior score, which means that the participants used the cartoon face as a cue to determine how much reward they would receive. Then, the object appeared in the correct position, and the patient navigated to that position for further learning. Behavioral events and motion data were written to log files with a temporal resolution of 10 ms. The patient was asked to complete more than 100 trials but could be instructed to pause or withdraw from the task at any time.

### Classification of goal objects and epochs of interest

The drop error in each trial is the difference between the correct location of the object and the location confirmed by the participant, as shown in Fig. 1B. For each participant, the mean value of drop error of each object in all trials he meets in the experiment can be obtained. According to the drop error, the eight goal objects of each subject were divided into two groups with equal numbers: a GoodPerf group with small drop errors and BadPerf group with large drop error. In other words, GoodPref group included all the trials of four objects with clear target reward memory, while BadPref group included all the trials of the other four objects with relatively vague target reward. Consistent with previous studies^15,17,18^, the subsequent analysis of stereotaxic EEG data focused on periods of fast movement during the retrieval phase. For each subject, fast movement is defined as the part where the speed order is in the first third of all movement time points of the subject.

### Intracranial EEG recordings and artifact removal

**Figure 2:**
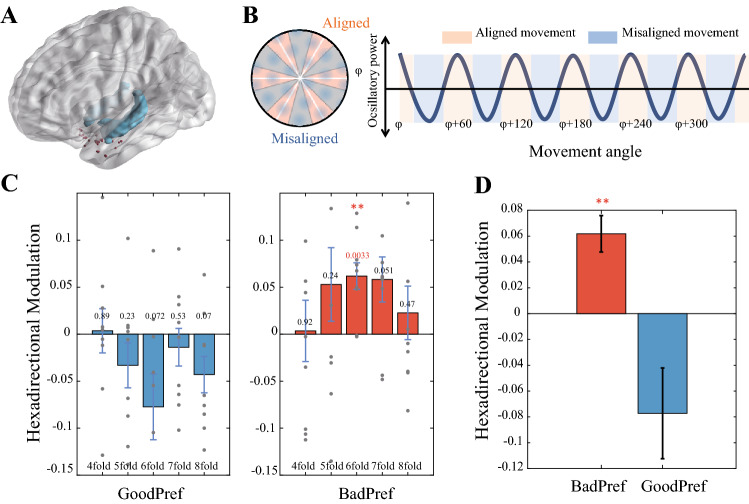
Hexadirectional modulation of entorhinal theta power by movement direction under the influence of goals. (**A**) Depiction of all the electrode contacts in the entorhinal cortex (red dots). (**B**) According to alignment or misalignment with the preference angle φ (left figure), we predicted the sixfold rotational symmetric sinusoidal modulation of the theta power signal by the moving direction according to the schematic diagram (right, adapted from Maidenbaum et al. (PNAS, 2018)). (**C**) GoodPerf and BadPerf groups correspond to each rotational symmetric modulation, among which only the sixfold modulation of the BadPerf was significant (*t* test, *p* = 0.003). (**D**) The BadPerf had significantly higher hexadirectional modulation than the GoodPerf (*t* test, *p* < 0.01).

Experimental data were collected at Yuquan Hospital affiliated with Tsinghua University, Beijing 301 Hospital, China, and the Epilepsy Department of Freiburg University, Germany. Our research programme was approved by the respective institutional review committees of the three hospitals. All patients provided written informed consent. The positions of all electrode contacts are shown in Fig. 2A, which was drawn using the BrainNet Viewer toolkit^19^. The stereotaxic EEG data sampling rate was 2000 Hz. An electrode contact near white matter was used as a reference. Twenty-five electrode contacts in the entorhinal cortex of 9 subjects were analyzed. No seizures were observed in any patients within 1 h before and after the trial. Interstitial spikes (IIS) and other artifacts were removed with an automatic cleanup program. When the envelope of the unfiltered signal was more than 4 standard deviations above the baseline calculated for the mean value of the whole signal, it was regarded as the artifact to be cleared.

### Time–frequency analysis

First, a notch filter was applied to all original data in the experiment recorded at 50 Hz and harmonic frequencies to eliminate power frequency interference. Next, all trials, including epileptic spikes and other artefacts, were excluded from further analysis. Then, consistent with a previous study^15^, all data were bandpass filtered in the theta frequency band (4–8 Hz). We first use FFT function to transform the data to frequency domain, then apply the Gaussian function with standard deviation of 0.7 Hz to attenuate at theta boundary frequency, and then use IFFT function in MATLAB to transform the data back to time domain. The Hilbert transform was used to extract the theta (4–8 Hz) power at each electrode channel. The power value was normalized to the average theta power of the electrode during all fast movement periods for subsequent analysis.

### Analysis of the hexadirectional modulation of theta power under each condition

In different datasets divided according to behavior, we analyzed the hexadirectional modulation of movement direction to theta power using a computational method described in previous studies^15,17,18^ (Fig. 2B). As mentioned above, according to the average drop error of different objects, the data were divided into GoodPerf and BadPerf. In the two groups, the dataset was divided into the boundary region and the central region according to the distance of their respective moving trajectories from the boundary. The corresponding theta power hexadirectional modulation index was calculated in different datasets divided according to their behavioral characteristics. The steps described below were performed. First, the recording time of EEG was aligned with the recording time of behavioral data. After extracting the fast movement fragments, the overall data were divided into six equally divided sessions in chronological order. We used the GLM model to model theta power and calculated φ as the preferred direction of movement related to the growth of theta power on half of the data (sessions 1, 3, 5). The GLM model contains two regressors: cos (6α) and sin (6α). The weights of the two regressors are obtained by regression, β_cos_, and β_sin_. Then, the preferred direction angle φ = [arctan (β_sin_/β_cos_)]/6 is calculated.

Again, using another GLM model, we tested whether theta power increased when the subjects moved along the previously obtained preference direction φ in the other half of the data (sessions 2, 4, and 6). In this model, the single regressor cos (6 (α − φ)) is used, and the number 6 in the factor indicates that the six-period rotation symmetry is tested. The resulting regression coefficient β_aligned quantifies the sixfold rotation symmetry of theta power by moving direction modulation. In previous studies, this coefficient has been referred to as the hexadirectional modulation indicator^15^ or ‘grid cell-like characterization’^18^. One β_aligned value was calculated at each electrode, and at the subject level, the β_aligned was averaged for all electrodes in the entorhinal cortex of that subject. Averaged β_aligned values were fed into second-level statistics across patients. Single-sample *t* tests were performed on β_aligned subjects to detect the presence of hexadirectional regulation of theta power in different datasets.

### Control analyses of different heading symmetry patterns

We conducted a control analysis on the rotation symmetry of the different cycles (4-, 5-, 7-, and 8-fold) of theta power in the entorhinal cortex, except for the sixfold modulation. Similar to the sixfold analysis, the two-step GLM model was used to complete the analysis. In the two GLM models, the constant 6 in the original model was replaced with a different number of cycles. Finally, the second level *t* test was conducted at the level of the subjects.

### Division of boundaries and central areas

The VR navigation environment is an arena with boundary blocking. We divided the field into the boundary area and the central area to compare the modulatory effects of conditions in different local environments on theta power. The specific operation is consistent with a previously reported method^15,18^. Two concentric circles with different radii were used to coordinate with the circular boundary of the arena and divide the arena into three navigation areas: the inner circle, the middle circle, and the outer circle. The basis of the radius setting is to balance the data number of trajectory points in different regions. The method used to calculate the hexadirectional modulation index β is consistent with the previous method: the preferred direction of movement is calculated from the data of the outer circle, and the preferred angle is determined in the data of the middle circle to obtain the hexadirectional modulation index β of the boundary part. The movement preference direction is calculated using the data of the middle circle, and the preferred direction is tested in the inner circle to obtain the hexadirectional modulation index β of the center part.

## Results

### Behavioral results

Preoperative patients with epilepsy (n = 9) completed the task in 45–70 min (mean ± SD, 59.4 ± 11.9 min), and more than 100 trials were completed. According to the average drop error of each object among the eight goal objects completed by each subject in all the trials, equal numbers of objects were divided into two groups: GoodPerf and BadPerf. We compared the behaviour and data balance of the two groups to avoid possible effects of deviations in the sample data on the subsequent calculations. The movement trajectory of the participants in the two object groups covered the whole test environment (the left column of Fig. 1C), a significant clustering of movement head direction was not observed (Rayleigh's test for non-uniformity, *p* > 0.05) (the right column of Fig. 1C), and the data capacity was balanced (*t* test, *p* > 0.05) (Fig. 1D). Participants showed significant differences in behavior between the two groups of objects (*t* test, *p* < 0.001) (Fig. 1E). In terms of time distribution, we investigated the time order of two kinds of trials: GoodPref and BadPref. Taking subject #1 as an example, we first observed the occurrence sequence of his two types of trials in the whole time of the experiment (Fig. 1F), and calculated the average sequence number of each type of trial as the trial serial number index. Finally, we compared the two groups of trial serial number indexes at the subject level, and there was no obvious difference in order (paired *t* test, *p* > 0.05) (Fig. 1G).

### Effects of an explicit goal location on hexadirectional modulation

**Figure 3:**
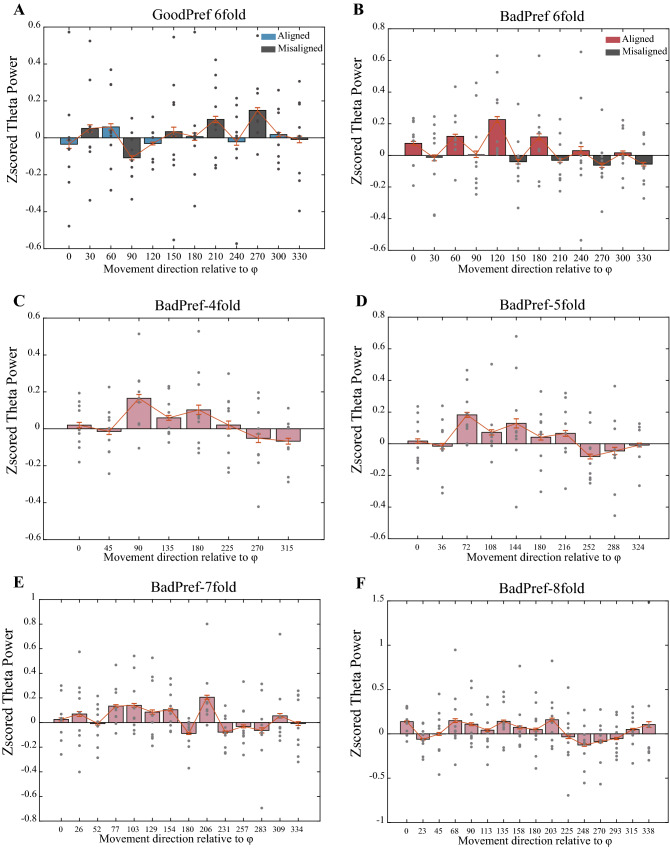
Theta power comparison in the case of movements aligned and misaligned with the grid axe of periodic symmetry. (**A**) Theta power of sixfold symmetry in GoodPref. (**B**) Theta power is higher during movements aligned with the grid axes as compared to misaligned movements. of sixfold symmetry in BadPref. (**C**–**F**) Theta power under other rotation symmetries (4/5/7/8-fold) in BadPref did not show the alternating trend of the high and low differences between aligned and misaligned.

We recorded stereotaxic EEG data (25 electrodes, as shown in Fig. 2A) from the entorhinal cortex of patients with epilepsy while conducting VR navigation behaviour experiments. Our analysis of the hexadirectional modulation of EEG signals focused on the theta band (4–8 Hz)^15,16^ and on the high-speed moving stage^15,18^. The method of analysing hexadirectional modulation using theta power is consistent with the previous EEG and fMRI methods for motion direction and signal hexadirectional rotational symmetry (see the “Methods”). We divided the data into GoodPerf and BadPerf groups according to the behavior of the subjects and calculated the strength of the hexadirectional modulation represented by theta power to analyse the effect of rewarding goal objects on the grid pattern. In the BadPerf group with no clear goal effect, we observed significant hexadirectional symmetry of the theta power modulation (*t* test, *p* < 0.01). In the control analysis of rotation symmetry, no other symmetric rotational modulations, such as 4-, 5-, 7-, and 8-fold (*t* test, *p* > 0.05) modulations, other than sixfold modulation were found (Fig. 2C, right panel). This result can also be directly observed from theta power comparison when moving along the aligned and misaligned directions (Fig. 3). Conversely, the hexadirectional modulation of theta power by the moving direction disappeared in the GoodPerf group influenced by the explicit goal (goal object whose reward location is clearly remembered by participant), and no other control modulation of the other type of symmetry type was observed (Fig. 2C, left panel). Hexadirectional modulations were significantly different between the two groups with or without clear goal effects (*t* test, *p* < 0.01, Fig. 2D).

### Spatial characteristics of goal-related hexadirectional modulation

**Figure 4:**
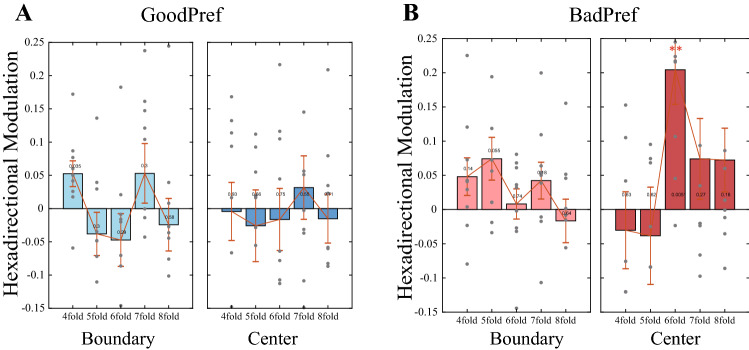
Spatial characteristics of the two groups of hexadirectional modulation. (**A**) All types of periodic rotation symmetries of the GoodPerf group are not significant in the space boundary region and the central region (*t* test, *p* > 0.05). (**B**) The BadPerf group shows no obvious directional modulation in the boundary region, but displays significant six-directional modulation in the central region (*t* test, *p* = 0.005).

Considering the effect of environmental boundaries on grid cell firing^20,21^ and because the effect of reward goals on grid patterns is often related to the specific location of goal objects^13,14^, we then refined the analysis in different subenvironments. We further analysed the data from the two groups with or without explicit goals by dividing the circular experimental environment into different ring-shaped feature regions (boundary area and the central area, see the “Methods”). We did not observe significant hexadirectional modulation of theta power in either the boundary or the central region in the GoodPerf group with a clear reward effect (*p* > 0.05, Fig. 4A). In the BadPerf group, which lacked a clear reward, the hexadirectional modulation of theta power was not observe in the boundary region (*p* > 0.05), while it was significant in the central region (*p* < 0.001, Fig. 4B). Thus, the spatial boundary anchoring effect of grid cell activity changes after the addition of nonspatial target reward factors, and the deformation of the grid pattern results from multiple factors.

**Figure 5:**
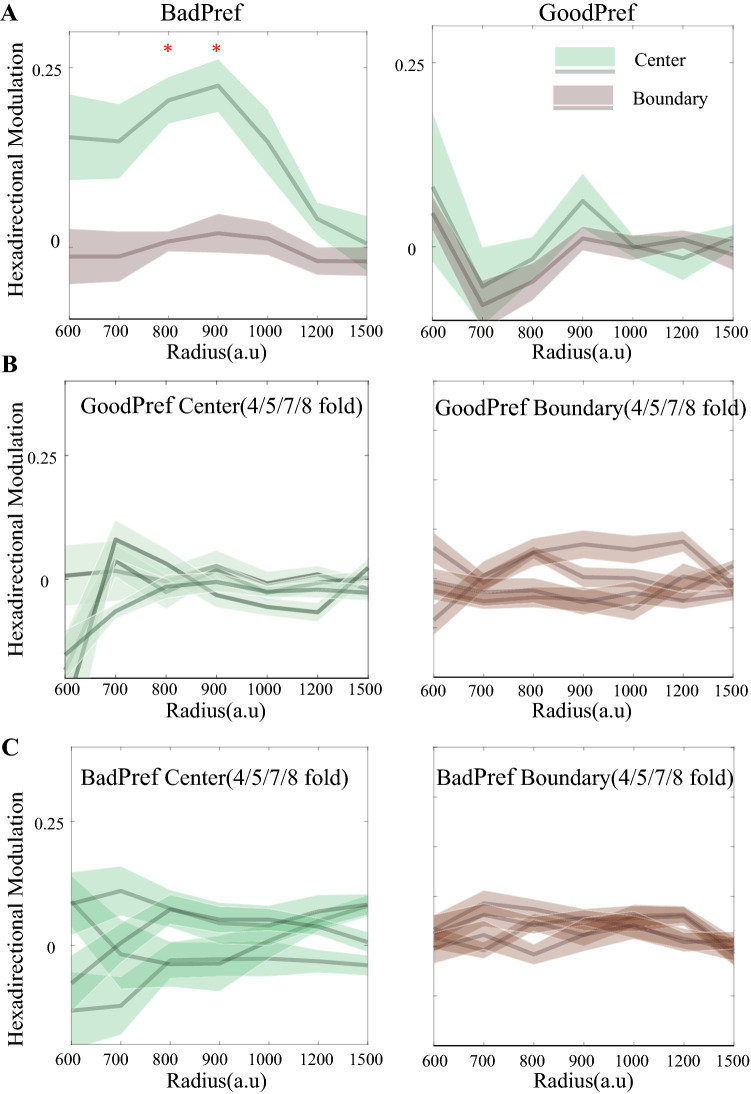
Analysis of symmetric rotational modulation using different partitioning radii. (**A**) For sixfold modulation, the BadPerf group shows a significant difference when the dividing radius of the boundary region and central region is 800–900 virtual units (*t* test, *p* < 0.001), while GoodPerf shows no significance for all the partition radius (*t* test, *p* > 0.05). (**B**) For the GoodPerf group, the modulation of other rotation symmetries (4/5/7/8-fold) is not significant (*t* test, *p* > 0.05) in the central area (left panel) and the boundary area (right panel). (**C**) For the Boodobj group, the directional modulation of the 4/5/7/8-fold rotational symmetry is also not significant (*t* test, *p* > 0.05) in the central area (left panel) and the boundary area (right panel).

Furthermore, we gradually changed the radius of the circle used to divide the subarea of the circular virtual arena and studied the changes in modulation under different divisions. In the GoodPerf group with clear rewards, no significant hexadirectional modulation was observed, regardless of how the dividing radius was changed (Fig. 5A, right panel). However, in the BadPerf group with no clear reward, the significance of hexadirectional modulation experienced a gradual presenting and vanishing process as the dividing radius changed from small to large (Fig. 5A, left panel). This evolving phenomenon is potentially explained by the unbalanced change in sample data. When the radius is too small, the amount of calculated data is insufficient due to the small amount of inner ring data. If the radius is too large, the data divided into the center circle include the data originally located in the boundary circle, resulting in the overlap of sample data. A stable and significant central region hexadirectional modulation phenomenon was only observed within the radius that equalized the data (*p* < 0.05, Fig. 5A, left panel). In both groups, any other control symmetry type was unaffected by the environmental division, with no significant modulation at the boundary or central regions (Fig. 5B, C). In summary, based on previous studies showing that the hexadirectional modulation of theta power reflects the neural characterization of grid cells from the level of mesoscopic oscillations, we provide additional evidence that human grid patterns are altered by nonspatial information. Based on the results of our study using humans, the firing pattern of grid cells is modulated by nonspatial information, and this effect is spatially localized, similar to rodents.

## Discussion

For many years, research on the spatial navigation system of the brain has focused on the objective perception of the natural world and physical space. The way humans and other animals behave suggests that they have reorganized their worldview and rediscovered the objective world by emphasizing information that they consider valuable. That is to say, the cognition of the objective world has an obvious subjective bias. The higher the organism is, the more complex the cognition and the more complex the nonspatial information that must be processed during navigation. In recent decades, place cells, grid cells, head direction cells, speed cells, and other navigation cells that encode spatial information have been discovered, but little is known about whether these cells represent spatial information and process nonspatial information at the same time. In particular, new evidence for grid cells has recently been obtained only in rat neuron recordings^13,14^. However, do human grid cells represent spatial information and organize and express memory? Is the hexadirectionally modulated oscillation signal in the entorhinal cortex not only affected by spatial position but also associated with empirical information in memory? Our research attempts to provide answers to these questions. Our work at the level of human intracranial electrophysiology reveals that the theta band neural oscillations in the entorhinal cortex carry not only the spatial information of hexadirectional modulated signals, but also the information of navigation reward. Our results provide a possible explanation for the effect of reward factors on the activity of grid cell populations, in which reward information will make the hexadirectional mode more changeable (such as destroying the boundary anchoring effect). However, the hexadirectional mode is relatively stable in the center area which has no clear reward, far away from the boundary and less affected. When repeatedly searching for objects in the same scene, the pattern of participants' grid cells in the same familiar circular arena should be regular. The difference is that for objects with more explicit reward information, there is a stronger disturbance in local space, thus disrupting the neural oscillation hexadirectional modulation at the population activity level. Next, we will further increase the comparative experimental research, such as adding scenarios without any navigation targets, and exploring the influence of reward factors in other geometric boundaries or even asymmetric boundary space environment. At the level of neural oscillation in the human brain, the role of the entorhinal cortex in nonspatial information annotation of navigation GPS maps has been identified, which supports the hypothesis that the brain's cognition of space and time has obvious subjective characteristics. Namely, the GPS navigation system of each person's brain will provide a personal positioning map composed of various factors.

In the future, this field of research will continue to be extended to the study of conceptual space, social space, and other abstract nonphysical spaces. The type of nonspatial information will be extended to other factors in addition to rewarding goals, such as risk and punishment. Studies examining the broad role of multimode information processing in the entorhinal cortex will help researchers understand remapping changes in grid cells caused by factors such as aging, neurological disease, or drug addiction. Furthermore, we will further explore the contribution of non-grid cells to the local field potential in the pattern of neuron population activity reflected by theta oscillations. The functions and roles of the entorhinal cortex in space and memory are gradually being recognized. Although grid cells have been identified as specific cells related to navigation and localization, their definition will be gradually rewritten: grid cells do not simply encode Euclidean space locations but rather the organization of memory under the comprehensive action of multiple factors.

## References

[1] Hafting T, Fyhn M, Molden S, Moser M-B, Moser EI (2005). Microstructure of a spatial map in the entorhinal cortex. Nature.

[2] Stemmler M, Mathis A, Herz AVM (2015). Connecting multiple spatial scales to decode the population activity of grid cells. Sci. Adv..

[3] McNaughton BL, Battaglia FP, Jensen O, Moser EI, Moser M-B (2006). Path integration and the neural basis of the ‘cognitive map’. Nat. Rev. Neurosci..

[4] Barry C, Hayman R, Burgess N, Jeffery KJ (2007). Experience-dependent rescaling of entorhinal grids. Nat. Neurosci..

[5] Derdikman D (2009). Fragmentation of grid cell maps in a multicompartment environment. Nat. Neurosci..

[6] Krupic J, Bauza M, Burton S, Barry C, O’Keefe J (2015). Grid cell symmetry is shaped by environmental geometry. Nature.

[7] Wernle T (2018). Integration of grid maps in merged environments. Nat. Neurosci..

[8] Constantinescu, A. O., OReilly, J. X. & Behrens, T. E. J. Organizing conceptual knowledge in humans with a gridlike code. *Science* **352**, 1464–1468 (2016). 10.1126/science.aaf0941 PMC5248972 27313047

[9] Horner AJ, Bisby JA, Zotow E, Bush D, Burgess N (2016). Grid-like processing of imagined navigation. Curr. Biol..

[10] Garvert, M. M., Dolan, R. J. & Behrens, T. E. A map of abstract relational knowledge in the human hippocampal–entorhinal cortex. *eLife* **6**, e17086 (2017). 10.7554/eLife.17086 PMC5407855 28448253

[11] Kaplan, R. & Friston, K. J. Entorhinal transformations in abstract frames of reference. *PLoS Biol* **17**, e3000230 (2019). 10.1371/journal.pbio.3000230 PMC6497227 31048835

[12] Epstein RA, Patai EZ, Julian JB, Spiers HJ (2017). The cognitive map in humans: Spatial navigation and beyond. Nat. Neurosci..

[13] Boccara CN, Nardin M, Stella F, O’Neill J, Csicsvari J (2019). The entorhinal cognitive map is attracted to goals. Science.

[14] Butler WN, Hardcastle K, Giocomo LM (2019). Remembered reward locations restructure entorhinal spatial maps. Science.

[15] Chen D (2018). Hexadirectional modulation of theta power in human entorhinal cortex during spatial navigation. Curr. Biol..

[16] Maidenbaum S, Miller J, Stein JM, Jacobs J (2018). Grid-like hexadirectional modulation of human entorhinal theta oscillations. Proc. Natl. Acad. Sci..

[17] Doeller CF, Barry C, Burgess N (2010). Evidence for grid cells in a human memory network. Nature.

[18] Kunz L (2015). Reduced grid-cell-like representations in adults at genetic risk for Alzheimer’s disease. Science.

[19] Xia, M., Wang, J. & He, Y. BrainNet Viewer: A network visualization tool for human–brain connectomics. *PLoS ONE* **8**, e68910 (2013). 10.1371/journal.pone.0068910 PMC3701683 23861951

[20] Hardcastle K, Ganguli S, Giocomo LM (2015). Environmental boundaries as an error correction mechanism for grid cells. Neuron.

[21] Hägglund M, Mørreaunet M, Moser M-B, Moser EI (2019). Grid-cell distortion along geometric borders. Curr. Biol..

